# Exploring the Social Trend Indications of Utilizing E-Commerce during and after COVID-19’s Hit

**DOI:** 10.3390/bs13010005

**Published:** 2022-12-21

**Authors:** Bahjat Fakieh, Ari Happonen

**Affiliations:** 1 Information Systems Department, King Abdulaziz University, Jeddah 21589, Saudi Arabia; 2 Software Engineering Department, LUT School of Engineering Science, LUT University, 53850 Lappeenranta, Finland

**Keywords:** COVID-19, digitalization, e-commerce, Egypt, Saudi Arabia, social trend, societal behavior, e-services, online, social distancing

## Abstract

COVID-19 is a major global crisis affecter, changing global norms and societal behavioral models. Many companies have faced existential crises, but on the other hand, businesses that were and are helping others to boost digitalization, ICT and software solutions deployment, remote communications integration, e-commerce & e-services, and so on, have boosted their businesses, as people shifted online during the global lockdown and international travel restrictions. Our work explores the trend of e-commerce and e-services utilization during the ease of restrictions and the social distancing period to forecast the trend continuation patterns after the pandemic. An online survey was conducted and targeted individuals in Saudi Arabia and Egypt, resulting in 155 participants. The data were analyzed from four perspectives: demographics, COVID-19 health impact, trend analysis, and regression analysis. The results indicate heavy utilization of e-commerce and e-services during the global movement restrictions and travel bans. This trend has, however, significantly reduced during the ease of restrictions and social distancing period. Utilizing e-commerce and e-services in Saudi Arabia and Egypt, based on the research data, is positively correlated to the outbreak conditions. On the other hand, current data still does not give clear indications, and this pattern is going to be mostly, partly, or not at all permanent now as societies are returning to mostly a free movement of people and marginally restricted social distancing times.

## 1. Introduction

The years 2020 to 2022 will remain a stand-out time in human history due to the outbreak of coronavirus [1,2,3] and the European energy crisis due to the Ukrainian War [4] that led to one of the fastest-phase periods of digitalization and boosted focus on sustainability [1,2,5] in different industrial, educational [6], municipal, and NGO contexts. Even the most traditional companies shifted their actions, too [7]. Historically, pandemics with similar big problems tied to them, like in the case of the COVID-19 outbreak, have happened before. These diseases have killed millions of people around the world, but none of them had as severe social distancing limitations. Examples of previous experiences with diseases include the smallpox outbreak in 430 B.C. and the spread of the Ebola virus in 2014.

During the early stages of the COVID-19 outbreak, the vision toward the virus was not consistent [8]. While some governments and individuals considered it to mostly resemble a fairly common virus, similar to the common flu, others took it differently and raised the warning about serious challenges the virus could bring to the table. However, the harsh reality was quite evident in 2020, and the entire world did acknowledge the seriousness of COVID-19 in terms of its rapid global spread and impact on individuals’ health, countries, continents, global societies, and economies [1,2].

According to the World Health Organization (WHO) and Worldometers, on 5 December 2022, severe waves of COVID-19 hit 220 countries, territories, and international conveyances, resulting in 650,126,812 confirmed infected people and 6,646,993 death cases [9,10]. This wave affected the global ecosystem and led to a change in habits of interacting and dealing with others [11,12]. Also, governments acted to set emergency regulations to contain the outbreak of the virus, such as social distancing, travel bans, and partial or complete lockdowns. This included restrictions leading to an ability for people to move outside, limiting movements between districts, and confinement of people to their own houses, without being able to go out [13,14]. This change pushed the pre-COVID-19 digital era transformation into high gear.

One of the major affected industries because of the pandemic is individuals’ trends toward e-commerce, which solved several logistic issues that assist human survival [15,16]. Thus, several research studies were conducted to explore the critical factors of e-commerce resilience [15,17] and the societal behaviors and trends toward e-commerce during and after the pandemic [16] in different industries and countries, such as food and beverage [18], retailing [19], fashion [20], and real estate industries [21]. However, such studies are limited in Middle Eastern countries to explore and predict the social trends of individuals toward utilizing e-commerce. This would be a barrier to answering significant questions related to the future of e-commerce and online business models in the Middle East, according to the behavioral changes after the pandemic hit. Therefore, this study aimed to explore the likelihood of the post-pandemic utilization of e-commerce by consumers. Saudi Arabia and Egypt were selected to conduct this study.

The following sections of this research paper contain a review of the global impact of COVID-19 that led to the research questions. Then, the research methodology is discussed before the analysis of the collected data. This analysis is divided into four stages: the demographical analysis, the health impact of COVID-19 on participants, the trend analysis toward utilizing e-commerce and e-services, and the regression analysis. After that, the results are discussed before the study is concluded, and limitations are highlighted to propose the future research direction and to guide researchers to fill the gap.

## 2. Literature Review

### 2.1. COVID-19 Global Impact

The global reaction of individuals and governments to control the rapid spread of COVID-19 resulted in several positive, neutral, and negative consequences, affecting multiple domains. Domains like, but are not limited to, local and international government policies [22,23], the economy [24,25,26], tourism [24,27], nature and the environment [28,29], society, psychological [30] and social behavior [31,32,33], health [34,35], and impact on food security [36,37]. As we can see, the consequences of COVID-19 affected both the local and international government policies in almost every country that tended to create new policies and/or modify or suspend current policies [22,23]. For example, if COVID-19 impacts are the enforcement of social distancing, the early travel ban, border closings, airport and seaport closings, strict COVID-19 testing, emergency laws, the allocation of emergency budgets [22,23], and the shifting online of some services and the previously highly face to face activity leaned [24] education system [38], crowd participation activities [39] changed the pre-COVID-19s business model innovation models [40] to new norms.

The outbreak of COVID-19 made the local and global economies less stable and demanded more trust in places [41] where natural trust (e.g., shared risk on supply chain development) between players was more evident in history and dynamics of supply chains have developed a lot lately. Several economies even ended up in a critical situation, such as loss of jobs, reduced business market values, and reduction in sales [26,42]. The educational sector was in crisis at the start of the COVID-19 pandemic. Globally, most school systems were not prepared to jump to a complete remote teaching model [43], but on the other hand, the crisis did generate a huge load of new educational innovations, too [44]. Additionally, the global stock markets and crude oil prices were severely hit [45,46]. On a positive note, some sectors were able to adjust fast, and they found significant business boost opportunities from their skills and resource utilization capability to reinvent their business models. This added to their business values and stocks. Good examples are technology business sectors, autonomous and robotized solutions providers, online services, medical, sports (especially individual activities, but also esports too [47,48]), and also some public services too.

The international tourism, travel, and people transportation services industries were the sectors most negatively affected by COVID-19 due to the lockdown, travel ban, and border closings [49,50]. The tourism industry was affected from the first day of the lockdown, as travelers, who are the main customers of this industry, suddenly stopped booking flights, trains, and hotels, renting cars, and engaging in entertainment activities [27] as some of the individuals shifted to the virtual tourism [51]. In addition, the COVID-19 crisis had a further severe impact on the travel industry when people started asking to cancel and receive refunds for existing bookings [27]. The continuous spread of the virus led to an extension of the travel ban in entire countries and even domestically, resulting in the historic bankruptcy announcements of well-known airports and airlines [52,53,54,55]. From the natural and environmental point of view, the effect of COVID-19 surprised the globe. For instance, as most people stayed at home during the lockdown, air quality improved noticeably in several contaminated areas, such as high-density cities in south and southeastern Asian countries [29]. In addition, a variety of wild and rare animals were spotted on city roads as they were enjoying the quiet environment of the lockdown and roaming for different purposes, such as looking for food [28].

The unexpected impact of COVID-19 on several domains included society and individuals’ social and psychological behavior, such as coping with social distancing [31,56]. This required distancing came with the tendency to stay at home to reduce the likelihood of being infected and virus transmission. However, some individuals decided to take the risk of interacting with others due to their panic about food availability or having less income by staying at home for a long period [57]. Reaching the required level of food security is one of the top global requirements for living [58]. Thus, COVID-19’s effect on this domain left some countries facing a critical challenge. The outbreak of COVID-19 and the aforementioned consequences resulted in severe food security impacts in several countries, such as Nepal [36], Kenya, and Uganda [37]. The sudden policies and restrictions led to a greater scarcity of food; workers, laborers, handcrafters, and farmers could not work as usual, while the restriction of movement prevented individuals from buying what they needed from other areas [36,37]. The issue of obtaining food from other areas was mitigated in several advanced countries by the use of different solutions, such as food delivery applications [58].

The mentioned global situation raises e-commerce and e-services market-related short and long-term effects and impacts the connected question, which is: What are the social behavior and demands of individuals toward utilizing e-commerce and services during and after the COVID-19 pandemic in Saudi Arabia and Egypt?

The next section will review the impact on e-commerce and e-service providers in Saudi Arabia and Egypt.

### 2.2. E-Commerce Market

The E-commerce market in the Middle East and North Africa (MENA) region is continuously evolving and grew by 35% in 2020 compared to the previous year. In more detail, the market value of e-commerce in MENA was equal to $11 billion in 2018. This number increased to $15 billion and $22 Billion in 2019 and 2020, respectively. The market is expected to reach $30 billion by the end of 2021 [59] as a result of different factors, including the spread of COVID-19 that led individuals to shop online rather than visiting stores physically [59,60,61], where the global digital transformation was boosted as a result of the pandemic [5]. Moreover, it is predicted that the value of e-commerce in the MENA region will reach $104.1 Billion and $183.4 by the end of 2022 and 2026, respectively [62]. Saudi Arabia, UAE, and Egypt are among the top players in leading this positive trend.

The market of e-commerce in Saudi Arabia is considered the 25th largest e-commerce market globally [63] and is one of the top e-commerce markets in the MENA region that offers a variety of e-commerce and services to consumers by having tens of online stores that compete to gain more from the market [64]. In 2020, Saudi Arabian e-commerce revenue increased by 34% compared to 2019. Namshi.com scored the highest rate in Saudi Arabia in net sales by $126 million. It is followed by jarir.com, extra.com, amazon.com (the Saudi division), and noon.com with net sales of $91 million, $64 million, $52 million, and $51 million respectively [65]. As the market could be affected by several factors and conditions, the forced lockdown played a significant role in boosting the Saudi market of e-commerce and services that led to a significant increment in the e-commerce and e-services market [60].

The Egyptian e-commerce and e-services market has the potential to be one of the main markets in the MENA region, where the number of internet users within the border of Egypt exceeded 53.5 million in 2019 [66]. This large number led to the rank of the Egyptian e-commerce market as the 48th largest market in the world [67]. Also, the Egyptian e-commerce and e-services market took a significant advantage in 2019 and 2020, which was affected by the global market condition and by the COVID-19 lockdown [61]. Considering the net sale in 2020, Souq.com (owned by Amazon.com) scored the top rank with total net sales of $116 million. It was followed by bthech.com, which scored second with net sales of $21 million. The next three largest e-commerce players in the Egyptian market were lcwaikiki.eg, elarabygroup.com, and hihonor.com, with net sales of $17 million, $13 million, and $12 million respectively [67].

Not only did Saudi Arabian and Egyptian markets gain from the pandemic condition, but the global market took advantage of the lockdown and social distancing. For instance, according to Yahoo Finance, the price of a single Amazon share in the stock market was $1847.84 on 31 December 2019, while the very high demand for buying products and services online boosted the stock price dramatically to $3499.12 by 1 September 2020 [68]. In addition, eBay’s stock price was investigated in the same period. It was valued at $36.11 and rose to $53.65 [69].

## 3. The Gap and Research Questions

So far, the reviewed literature has shown a considerable effect on individuals’ daily lives due to the impact of COVID-19. However, as already mentioned, some industries were able to adjust quite fast, and those industries truly took advantage of the many consequences of the outbreak. Information and communication technology (ICT), warehousing [70], logistics [71], and supply chain-related companies are among the sectors that gained the opportunities to make significant changes and gain profits from their activity during the pandemic. Interestingly, Digital citizen science projects [72] or community-based monitoring activities [39] did not seem to jump to new levels, even though social distancing could have given people a lot more time to contribute and digitalization had provided tools to do so. Additionally, several industries, such as IT business values, stocks, and food delivery companies, did skyrocket, and they found new, never before realized customer sectors [68,69,73].

Remaining at this high position in the market after the crisis requires considering several factors in terms of business operations and future plans. Porter’s Five Forces model emphasizes five main factors to maintain one’s business position in the market. Those five factors are potential entrants, the power of the suppliers, the power of the buyers, industry competitors, and the power of the substitutes [74]. Therefore, the impact that came from the power of the buyer presented a high demand in some online commerce and e-services when several other sectors suffered severely from the crisis and many announced bankruptcies. Thus, it would be better to investigate the possible social trend that would affect enterprises’ strategic and tactical decisions on the national and international scopes. Therefore, some research studies highlighted this issue from several perspectives, such as comparing consumer trends before and after, but not during, the pandemic [75,76] or focusing on some specific industries, like exporting services [77] and the healthcare industry [78]. However, there is a lack of research highlighting the possible social trend toward utilizing online services and commerce in Saudi Arabia and Egypt as two of the main online markets in the Middle East, as the current studies were limitedly explored. Also, the other side of the gap would be presented in the current studies by focusing on the periods before and after the pandemic, as mentioned before, or only during the COVID-19 period, but not on exploring the shift in consumer preferences during and after the pandemic in the Middle East. This briefly described gap led to the development of the main research question, which is as follows:

- What are the social behavior and demands of individuals toward utilizing e-commerce and services during and after the COVID-19 pandemic in Saudi Arabia and Egypt?

Answering this question will provide more insights and give the ability to build a hypothesis for a large-scale study.

## 4. Methodology

To answer the main research question and to understand the possible social behavior of utilizing e-commerce and services after the COVID-19 period. The methodology of this study was started by analyzing the current literature, which was followed by collecting the necessary data. Then, a statistical analysis was conducted. This stage consists of four stages. The demographic analysis comes first to understand the nature of the sample. Then, the health impact of the pandemic was explored on the sample to find the possible relationship between the sample health record and their behaviors toward e-commerce utilization. The third stage explored the status of consumers’ online purchasing and their future likelihood trend in the e-commerce industry, while the fourth stage focused on the regression analysis to get more insights into the relationship between the COVID-19 health record and consumer trends in e-commerce.

**Figure 1:**
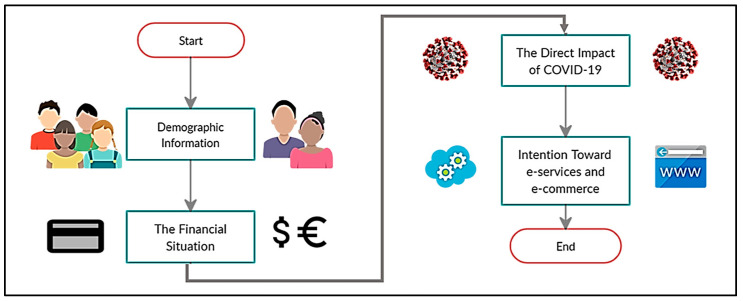
The Sections of the Conducted Survey.

Collecting data was essential to obtain insights. Thus, an online survey was created to obtain the required data. The sampling method followed the snowball sampling technique, where each participant was asked to invite others to participate. The data was collected via multiple sources by utilizing a semi-structured online survey [79,80]. This survey consists of four sections, as illustrated in Figure 1. This survey was mainly in Arabic, the main language in the investigated countries. Thus, a brief description of the study and a consent statement was written at the beginning of the online survey in Arabic and English to ensure a complete understanding of the main aim and the research-based intention in using and analyzing the collected data, as well as to give participants the complete rights and freedom to accept or reject the invitation to be part of the study.

The first section of the survey focuses on demographic information, i.e., independent variables such as age, gender, and marital status. The following second section highlights factors that could describe the financial situation of the participant. The second section also provides independent variables that would affect decisions about post-COVID-19 trends in utilizing e-services and e-commerce. Employment status and monthly income are the factors explored in the second section.

The third section investigates the direct impact of COVID-19 on participants from two perspectives: the stay-at-home orders during the peak periods of COVID-19 and whether the participants or any of the people surrounding them were infected with COVID-19. In direct connection to the issue, the fourth section investigates the participants’—the consumers—intention toward e-services and e-commerce in four timeframes. These timeframes are before the outbreak of COVID-19, during the peak of the pandemic and the highest movement restrictions (including the curfew), after the partial ease of the governments’ restrictions, and after the COVID-19 stage, when the world gets proper and complete medicines and protections against the pandemic.

To distribute the developed survey, a professional survey distribution organization was contracted to distribute, collect, and manage the process. This process included the following stages:

- Selecting the correct sample, such as the geographical location and the age;
- Preparing data for analysis at the preprocessing stage. This included removing incomplete records and incorrect entries.

After that, data were prepared for analysis. The following section will analyze and present the results of the conducted study.

## 5. Data Analysis and Results

The conducted survey was distributed in two Middle Eastern countries: Saudi Arabia and Egypt. The invitation to participate in the questionnaire was sent via social media with the help of a hired marketing specialist. Thus, the convenience sampling methodology was used to collect the required data [81], which resulted in a sample size of 155 responses.

The analysis of the collected data went through four main stages. The first stage was the implementation of demographic analysis to view the main statistics and obtain the first insights. This stage covers the first two sections of the conducted survey, illustrated in Figure 1. The second analysis stage highlights the direct health effects to conduct a regression analysis that investigates the relationship of several factors that could affect online consumer behavior toward e-commerce and e-services. The third stage exposed the trend of utilizing e-commerce and e-services in four timeframes, as mentioned in the methodology section. The fourth analysis phase investigated the relationship among variables. For the findings of these stages, the detailed analysis and the obtained results are discussed in detail in the discussion section at the end of this manuscript.

### 5.1. Demographic Analysis

To understand the nature of the participants, Table 1 shows the demographic information and the independent variables of the collected data. The data, as shown in Table 1, presents the 155 people who participated in the data collection process: 104 (67%) from Saudi Arabia and 51 (33%) from Egypt. With regard to age groups, the participants were divided into five groups: 18–30, 31–40, 41–50, 51–60, and 61+. However, the analysis revealed that there was only one participant older than 60 years. Therefore, the last two groups were merged into one group to cover participants older than 51. The table also shows the approximate similarity between the participating genders. The sample contains 82 (53%) males and 73 (47%) females, while, in terms of marital status, the sample consists of 84 (54%) single participants, 69 (45%) married participants, and only two (1%) divorced participants.

With regard to employment status, the study divided participants into five groups. The majority were unemployed: 63 (41%). The second highest group of 42 (27%) participants were employed in the private sector. The third highest group was participants employed in the government sector: 24 (15%). This was followed by students and business owners: 14 (9%) and 12 (8%), respectively.

To gain more insights into the financial situations of the participants, Table 1 is used to reveal that the obvious majority of 107 (69%) of the participants were earning less than 10,000 every month in the local currency; those currencies are the Saudi Riyal (SAR) and the Egyptian Pound (EGP). The main reason for not calculating the income using a unified currency is to consider the different costs of living in both countries. In addition, about 46% of the studied sample’s monthly income was negatively affected by COVID-19, while for 52%, it was not affected. Only 3% found more opportunities to raise their income during COVID-19.

Lastly, nearly half of the participants (45%) were suffering from full-day curfews that the governments ordered to contain the outbreak of COVID-19. The following subsection discusses the trends of utilizing e-commerce and services by the studied sample.

### 5.2. Health Impact of COVID-19

The impact of COVID-19 affected the behavior and the future willingness to utilize e-commerce and e-services. Thus, every participant was asked if they, their families, or their relatives had been infected with, or had died from, COVID-19.

It is shown in Table 2 that 88 (57%) participants were not infected by the outbreak of COVID-19. This leads to the conclusion that 43% were affected directly or indirectly by the infection of the virus. In detail, 17 (11%) participants, 33 (21%) of their families, and 42 (27%) of their relatives and friends were infected with COVID-19. Also, 5 (3%) of participants said that their family members had been killed by COVID-19, and, surprisingly, 39 (25%) reported that their relatives and friends had died of the disease.

Note: The phrase “at least…” in the statistics of the above paragraph is mentioned as the participants were asked whether or not they had experienced cases of COVID-19. A “yes” answer indicates that they would have experienced one or more cases of COVID-19.

### 5.3. Trend Analysis

The initial step of exploring the participants’ views toward utilizing e-commerce and services was dedicated to exploring the general intention of utilization (Table 3). Four time periods are used: before COVID-19, during the peak of the virus (which included the curfew and the restriction of movement and travel), after the easing of restrictions with the enforcement of social distancing), and after the virus. The table shows a slight increase in utilizing e-commerce and services during the outbreak from the first stage. This would indicate that the majority of stage 2’s customers were the same customers as those before the outbreak.

Participants who did not utilize the investigated services in the first two stages were asked for an explanation for their decisions. These were a desire to see and touch the products physically before buying them so as to feel comfortable and ensure the intended quality, the preference for going outdoors, the trust level toward the offered products and service providers, and late delivery. Also, the intention toward e-commerce and e-services decreased a little with the ease of the movement restriction, to be 0.57 instead of 0.63 in stage 2. Although the difference between the stages was not significant, participants were asked about the reason for this reaction. The intention to go outdoors with care and to feel free was the main reason.

The information presented in Table 3 gives general indications of utilizing e-commerce and e-services by individuals in the four explored periods. However, it was expected that some participants did not consider specific activities while surfing the web, such as utilizing e-commerce and e-services activities. Therefore, Table 4 presents detailed information as a result of asking each participant.

**Figure 2:**
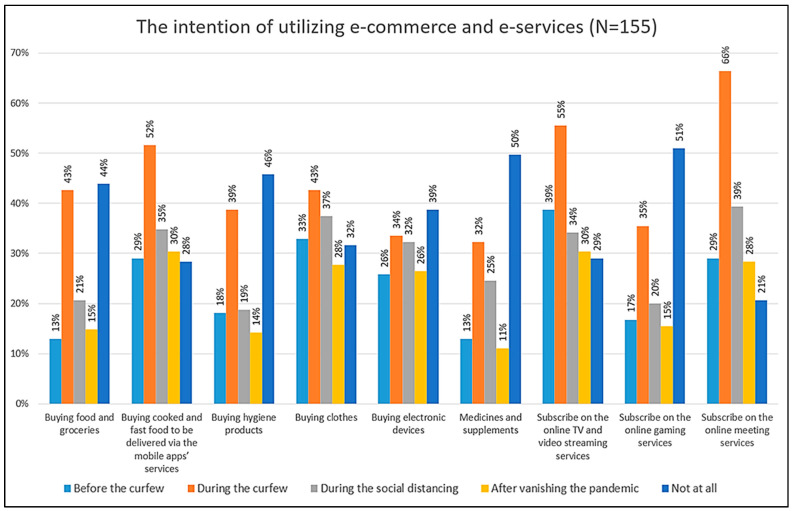
The intention of utilizing e-commerce and e-services (*n* = 155).

The percentage of those details is illustrated to be readable in Figure 2. Regardless of the last column of each category (dark blue), which shows participants who do not like to utilize the examined products and services, the busiest period was during the curfew. However, the figure gives a negative indication of the future behavior of utilizing e-commerce and e-services. Most of the utilized e-commerce products and services returned to normal situations as before the outbreak of COVID-19. Additionally, the results show that utilization of some products or services is expected to be even lower than the normal trend when one compares the periods before the outbreak and after the virus, such as subscribing to online TV and video streaming services.

Regarding the results in Figure 2, buying food and groceries online was the trend among 43% of the participants during the curfew compared to only 13% before the outbreak. This number declined by about half, to 21%, after the easing of movement restrictions. In addition, participants are aiming to reduce their online buying to only 15% after the end of the pandemic.

The trend of buying cooked and fast food online and taking advantage of delivery applications was admitted to by 29% before the outbreak. This trend jumped to 52% during the curfew and went back to 35% during the social distancing period, then to the expected 28% after COVID-19.

Buying hygiene products was also affected clearly by the hit of COVID-19. The increase in the utilization ratio from 18% before the outbreak to 39% during the curfew proves this claim. Again, this number declined to 19% during the ease of the restriction period and is expected to go down to 14% after the pandemic ends.

Another example is the trend towards subscribing to online meeting services for personal purposes. The restriction of movement led to a skyrocket in the utilization ratio, from 29% before the pandemic to 66% during the curfew, which was the highest utilized category in the figure. This ratio went back to 39% during the social distancing period and is expected to decline to 21% after the pandemic.

The next subsection will run a regression analysis to explore relationships between the factors that would affect the trend of utilizing e-commerce and e-services.

### 5.4. Regression Analysis

The main objective of a regression analysis is to explore the possible effect of COVID-19 infection on the participants’ decisions to utilize e-commerce and e-services during the social distancing period and after COVID-19. For the analysis, the examined variables are presented in Table 5. The independent variables were classified into two main categories: infection with COVID-19 and death caused by COVID-19.

In the first category, participants were divided into four groups according to the closest infection case to the participant. The first group contains participants who were infected directly by the virus. The second group contains people who were not infected but whose family members were. The third group includes participants who did not suffer from COVID-19, and nor did their families, but whose relatives or friends did. The last group contains people who did not encounter COVID-19 cases in their inner circle of family, friends, or relatives.

The second category explores the effect of deaths resulting from COVID-19 in terms of three groups. The first group contains participants whose family members have died of COVID-19. The second group includes participants whose relatives or friends died of COVID-19. The third group contains participants who had encountered any death cases due to COVID-19.

The presented data and its analysis show that the relationship between the intentions of utilizing e-commerce and e-services during the social distancing and after-COVID-19 periods is loosely coupled, where all *p*-values are above 0.05. Thus, the null hypothesis is accepted for all cases in which none of the virus infections or death cases would affect participants’ intentions.

## 6. Theoretical and Practical Implications

### 6.1. Principal Findings

The collected sample covered individuals from Saudi Arabia and Egypt. More than half of the sample was aged between 18–30, and the sample was approximately balanced between males and females.

More than half of the collected sample was not infected by COVID-19, and the other half were either infected or related to infected people. In addition, more than a quarter of the sample encountered death cases because of COVID-19 in their families or relatives.

The results of the trend analysis Section 5.3 were clearly in favor of the claimed significant direction toward e-commerce and e-services by the public, which led to the skyrocketing of stock prices for companies in this sector [68,69]. However, the result raised some concerns regarding the future status of those companies. As the consumer is considered one of the five main forces that Porter highlighted as their effect on the rivalry of businesses in the market [74], the intention of the majority of participants indicates that the utilization of e-commerce and e-services at this high rate would not continue after the pandemic ends. Although it was discovered in the literature that market research predicted the rise of e-commerce in Saudi Arabia in the coming years [65], this study is distinct in highlighting some alerts conducted in considering the post-pandemic of local consumer trends, including the drivers and challenges. This could open the doors for expanded scientific and market research to consider the highlighted factors on a larger scale to facilitate the promising future of e-commerce industry in the Middle East, especially in Saudi Arabia and Egypt.

### 6.2. The Effect of COVID-19

Before the regression analysis was conducted, it was expected that there would be a limited relationship between the health effect of COVID-19 and the psychological intention of utilizing e-commerce and e-services. However, the presented results in Table 5 exceed the expectation by neglecting the effect of the virus on the intention level of utilizing e-commerce and e-services completely. This would indicate that though individuals adapted to the online norm to process different daily tasks and businesses, people might be eager to return to pre-COVID-19 behavior after the pandemic ends, which unfortunately does not seems to do so, at least before 2023 [82]. However, there are several questions that should be considered in further detailed research, such as how fast individuals might return to the pre-COVID period. Are they going to return immediately or gradually? Do individuals need to keep some of their new online skills or avoid them completely? What is the proper date to consider as the starting point of the post-pandemic era?

## 7. Conclusions

COVID-19 is considered to be one of the most significant historical pandemics so far. It is the first pandemic that stopped the world as we know it, especially in travel and social entertainment, and recreational contexts, which ended up transforming individuals’ ways of living, at least for the time being, but in many permanent ways, as some already have predicted. The pandemic has “forced” nations and the global population to do fast-phased industrial digitalization and engage in the societal digital transformation of daily lives, the utilization of social communication channels, and change the way companies do their business. Still, some of the areas of digitalization did not jump into higher gear, like mass-customized products (e.g., 3D printed personalized solutions ordered from the internet and delivered straight to home). Nevertheless, the advancement of information and communication technologies led to the offering of several products and services via online channels and generated great shortages of physical devices needed for online channels, like webcams, mobile internet access modems, computers/tablets, etc., in general. This push towards a robust online presence helped provide most nations with the essentials of living, and individuals relied on e-commerce and e-services heavily during the outbreak.

This study explored the social trend of utilizing e-commerce and services during the easing of restrictions and social distancing as well as during the expected stage after the end of the pandemic. The results indicated that this exceptional trend toward e-commerce and e-services is expected to shrink after the normal situation returns. In addition, the results have shown that the lockdown period did change habits during the pandemic, and it is necessary to explore the facts of how this change would last after the pandemic, particularly if the post-pandemic period is like the pre-pandemic period, where people claim that they would want to return to the old norms and habits, as they are waiting for the end of the pandemic so that they can return to their “normal” lives. However, the reality is that many companies have changed their business models and operational practices, and they will never return to being exactly as they were in pre-pandemic times. This means there will not be a pre-pandemic “normal” available for people, no matter what they want.

Business organizations, owners, and economists should consider the expected hope of the customers to return to pre-COVID-19 norms, which could mean a second wave of needs to adapt again with their business models to keep up with the behavior changes of their customer base and to avoid any unexpected issues, such as the availability of products and services on-premises, and the possible impact on the global stock markets.

## 8. Limitations

This research study encountered several limitations. Although the studied sample consists of 155 individuals from two countries, it is recommended to conduct similar studies on wider ranges. Also, researchers should consider conducting similar studies in different countries, especially from the advanced world, as the results would help to create a comparison of the effect of the virus on several regions globally.

Additionally, as the trend analysis presented the limited effect of COVID-19 on the individuals’ intention toward e-commerce and e-services, it was planned to conduct a regression analysis to examine the effect on each country individually. However, dividing the participants would limit the sample size in each country and might lead to an unreliable result. Thus, it is recommended again that similar studies be conducted to focus on other demographic variables.

### Future Work

The limitations that were discussed in this study led to the proposal of a new project aiming to develop research hypotheses to forecast the future global trend of societies toward e-commerce and e-services. The proposed sample size would exceed 10,000 to synthesize the results.

## Figures and Tables

**Table 1:** Respondent demographics (*n* = 155 for each category).

Demographics and Variable	Frequency *n*, (%)
**Country**	
Saudi Arabia	104, (67%)
Egypt	51, (33%)
**Age (Years)**	
18–30	91, (59%)
31–40	45, (29%)
41–50	14, (9%)
≥51	5, (3%)
**Gender**	
Male	82, (53%)
Female	73, (47%)
**Marital Status**	
Single	84, (54%)
Married	69, (45%)
Divorced	2, (1%)
**Employment Status**	
Student	14, (9%)
Employed—Government Sector	24, (15%)
Employed—Private Sector	42, (27%)
Business Owner	12, (8%)
Unemployed	63, (41%)
**Monthly Income in the Local Currency**	
Less than 10 K	107, (69%)
From 10 k to 20 K	32, (21%)
More than 20 K	16, (10%)
**Change in Monthly Income Because of COVID-19**	
Reduced	71, (46%)
Increased	4, (3%)
Remained the Same	80, (52%)
**Highest Hourly Curfew Duration per Day**	
¼ of the day (0 to 6 h)	7, (5%)
½ of the day (>6 to 12 h)	51, (33%)
¾ of the day (>12 to 18 h)	27, (17%)
Most of or the entire day (>18 to 24 h)	70, (45%)

**Table 2:** Infection and death rate among participants because of COVID-19 (*n* = 155 for each row).

Infection/Death by COVID-19	Frequency * *n*, (%)
**Infection by COVID-19**	
No	88, (57%)
Yes—Me	17, (11%)
Yes—My Family	33, (21%)
Yes—Relatives or Friends	42, (27%)
**Death by COVID-19**	
No	115, (74%)
Yes—My Family	5, (3%)
Yes—Relatives or Friends	39, (25%)

* Please note that each row would take a value from 0 (0%) to 155 (100%), as some participants could be infected PLUS have some of their families, relatives, or friends infected as well.

**Table 3:** The utilization of e-commerce and e-services in four periods.

Stage No.	Time Period of COVID-19	Average *	Standard Deviation *
1	Before the pandemic	0.55	0.26
2	During the outbreak and the curfew	0.63	0.28
3	During the ease and the social distancing	0.57	0.36
4	After the pandemic	0.60	0.34

* All of the presented values were scaled from 0 (the lowest) to 1 (the highest).

**Table 4:** The intention of utilizing e-commerce and e-services (*n* = 155).

Activity No.	Online Activity	Frequency * *n*, (%)				
		Before the Pandemic	During the Outbreak and Curfew	During the Ease and Social Distancing	After the Pandemic	Never Like
1	Buying food and groceries	20, (13%)	66, (43%)	32, (21%)	23, (15%)	68, (44%)
2	Buying cooked and fast food to be delivered via mobile app services	45, (29%)	80, (52%)	54, (35%)	47, (30%)	44, (28%)
3	Buying hygiene products	28, (18%)	60, (39%)	29, (19%)	22, (14%)	71, (46%)
4	Buying clothes	51, (33%)	66, (43%)	58, (37%)	43, (28%)	49, (32%)
5	Buying electronic devices	40, (26%)	52, (34%)	50, (32%)	41, (26%)	60, (39%)
6	Buying medicines and supplements	20, (13%)	50, (32%)	38, (25%)	17, (11%)	77, (50%)
7	Subscribing to online TV and video streaming services	60, (39%)	86, (55%)	53, (34%)	47, (30%)	45, (29%)
8	Subscribing to online gaming services	26, (17%)	55, (35%)	31, (20%)	24, (15%)	79, (51%)
9	Subscribing to online meeting services	45, (29%)	103, (66%)	61, (39%)	44, (28%)	32, (21%)

* Please note that each row would take a value from 0 (0%) to 155 (100%).

**Table 5:** The effect of COVID-19 on participants’ trends toward e-commerce and e-services.

COVID-19 Effect	# of Groups	During the Social Distancing Period		After COVID-19	
		F Value (df)	*p* Value	F Value (df)	*p* Value
Infection	4	0.003 _(3, 151)_	0.955	0.112 _(3, 151)_	0.739
Death	3	0.111 _(2, 152)_	0.739	0.035 _(2, 152)_	0.852

Please note that # means “number” and df indicates the degrees of freedom.

## Data Availability

Data will be available upon request.

## Abbreviations

COVID-19	Corona Virus Disease 2019
E-commerce	Electronic commerce
E-services	Electronic services
EGP	Egyptian Pound (the currency of Egypt)
ICT	Information and communication technology
MENA	Middle East and North Africa
NGO	Non-Governmental Organization
RRT	Renal Replacement Therapy
SAR	Saudi Riyal (the currency of Saudi Arabia)
UAE	United Arab Emirates
WHO	World Health Organization
